# Global, regional, and national burdens of Urolithiasis in adolescents and young adults aged 10–24 years from 1990 to 2021: a trend analysis based on the Global Burden of Disease Study 2021

**DOI:** 10.3389/fruro.2025.1643340

**Published:** 2025-12-19

**Authors:** XinXin Wang, XiuWang Wei, JianBo Liang, YangYang Xu, DaMing Yang, XiuJia Wang, HuanWen Huang, ChangSheng Chen, KaiQiang Li

**Affiliations:** 1 Department of Urology, Guangxi Academy of Medical Sciences, The People’s Hospital of Guangxi Zhuang Autonomous Region, Nanning, China; 2 Department of Urology, The Second Affiliated Hospital, School of Medicine, Zhejiang University, Hangzhou, China

**Keywords:** global, adolescents and young adults, urolithiasis, disability-adjusted life-years (DALYs), incidence, mortality, burden of disease

## Abstract

**Background:**

Urolithiasis poses a significant health risk to adolescents worldwide, yet information on its burden and trends is limited. This study analyzed the evolving patterns of urolithiasis among 10-24-year-olds globally, regionally, and nationally from 1990 to 2021.

**Methods:**

This study predicted the global burden of diseases and investigated urolithiasis incidence and disability-adjusted life-years (DALYs) in 10-24-year-olds. We reported cases, rates per 100,000, and AAPCs globally, regionally, and nationally. We explored trends across age groups, sexes, and SDI categories, using Joinpoint regression to identify the year with the most significant global trend shift.

**Results:**

The global incidence of urolithiasis among adolescents and young adults aged 10 – 24 years has increased modestly, from 321.5 per 100,000 in 1990 to 342.6 per 100,000 in 2021, with an average annual percent change (AAPC) of 0.2. A significant upward shift was observed in 2009. Regionally, Tropical Latin America saw the largest increase, with a rise from 155.1 per 100,000 in 1990 to 296.4 per 100,000 in 2021, and an AAPC of 2.07. In contrast, East Asia experienced the most significant decline, dropping from 214.2 per 100,000 in 1990 to 140.2 per 100,000 in 2021, with an AAPC of -1.37. Nationally, Brazil showed the highest increase, with an AAPC of 2.14, while the Russian Federation had the highest incidence in 2021, at 812.7 per 100,000. The middle-SDI quintile countries saw the largest increase in incidence, with an AAPC of 0.45. However, countries with high to middle and high SDI scores demonstrated a decrease in incidence. From 1990 to 2021, the incidence of urolithiasis increased more rapidly among females than males, with an AAPC of 0.26 and 0.16 respectively. By 2021, there were 6,467,487 cases globally, 57.8% of which were in males. The most significant increase in incidence was observed among those aged 20 – 24 years, with an AAPC of 0.29.

**Conclusions:**

The global burden of urolithiasis in adolescents and youth is a significant health issue requiring international collaboration for better management. Enhancing diagnostic tools and implementing effective prevention and treatment methods are crucial.

## Introduction

Urolithiasis is a prevalent urological condition posing a significant burden on healthcare systems globally. The USA has witnessed a rising prevalence of urolithiasis over the past 30 years, projecting an additional economic burden of USD 24 billion per year by 2030 (1). While urolithiasis showed a declining tendency in the global burden of urological diseases in 2021 (2), pediatric urolithiasis remains an underestimated issue, occurring at a rate of approximately 10% of that observed in adults. Incidental discoveries due to nonspecific symptoms may lead to an underestimation of the actual incidence in children. Studies indicate that urolithiasis accounts for 1 in 1000 to 1 in 7500 pediatric hospital admissions (3). However, superior national data from resource-limited countries is lacking, making it difficult to ascertain the true extent of urolithiasis in adolescents and young adults in specific countries and vulnerable groups. In this study, we hypothesized an increase in the incidence of urolithiasis among individuals aged 10 – 24 years between 1990 and 2021. Our research aimed to investigate the worldwide patterns of urolithiasis, examining its incidence, disability-adjusted life years (DALYs), prevalence, and mortality rates at ten-year intervals beginning in 1990. We also aimed to identify the year with the most significant alterations in trends for these measures, stratify the global tendency by age category, sex, and sociodemographic index (SDI), and report trends at both the regional and national levels.

## Methods

In this study, we analyzed data from the Global Burden of Disease Study 2021, which collected periodic intersecting data on 369 diseases and injuries across 204 countries and regions from 1990 to 2021, including urolithiasis. We focused on urolithiasis data stratified by sex and age groups (10-14, 15-19, and 20 – 24 years) in 21 geographically contiguous and epidemiologically comparable regional clusters as defined by the GBD initiative. Adolescence, defined by the World Health Organization as the age range of 10 – 19 years, was categorized into younger adolescents (10 – 14 years), older adolescents (15 – 19 years), and youths (20 – 24 years) in this study to provide a detailed understanding of growth patterns during this developmental phase (4, 5).

## Results

### Global trends

**Figure 1:**
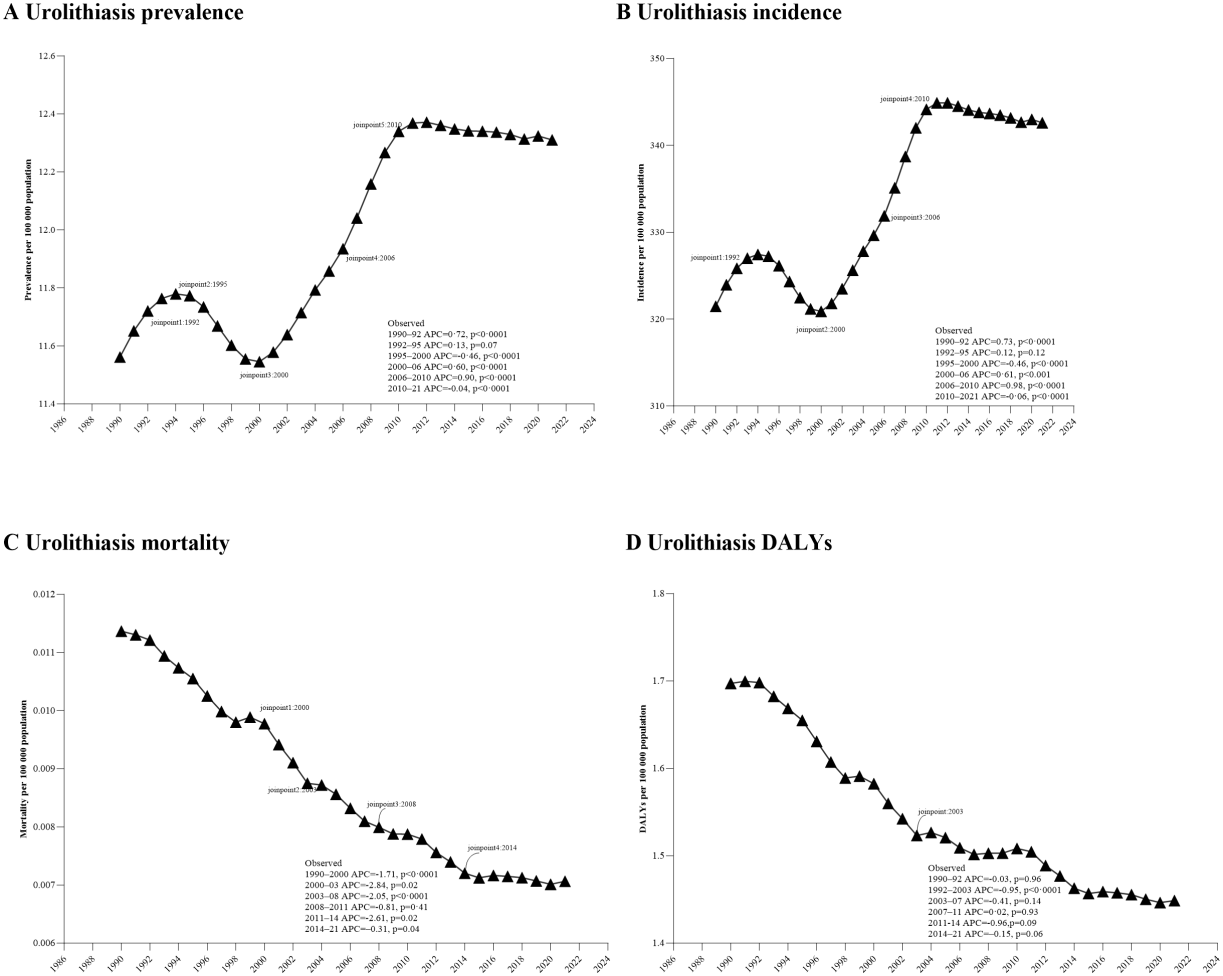
Joinpoint regression analysis of global Urolithiasis prevalence **(A)**, incidence **(B)**, mortality **(C)**, and DALYs **(D)** in adolescents and young adults aged 10–24 years from 1990 to 2021. APC, annual percentage change; DALYs, disability-adjusted life-year.

The incidence of urolithiasis among adolescents and youths globally increased between 2000 and 2009 (AAPC: 0.73 [95% CI 0.7 to 0.76]) and slightly declined between 2010 and 2021 (AAPC: 0.06 [95% CI: -0.05 to 0.07]) (**Table 1**). In 2021, the incidence rate was 342.6 per 100,000 population, higher than in 1990 (321.5 per 100,000). Similar trends were observed in prevalence. However, mortality and DALYs related to urolithiasis decreased between 1990 and 2021. Significant changes in incidence were identified in 1992, 1995, 2000, 2006, and 2010 (**Figure 1**).

**Table 1:** Global AAPCs in prevalence, incidence, mortality, and DALYs of urolithiasis.

Parameter	Mortality	p Value	DALYs	p Value	Incidence	p Value	Prevalence	p Value
	AAPC(95%CI)		AAPC(95%CI)		AAPC(95%CI)		AAPC(95%CI)	
Year								
1990-2021	-1.56 (-1.85,-1.26)	<0.01	-0.51 (-0.67,-0.35)	<0.01	0.2 (0.18,0.23)	<0.01	0.2 (0.18,0.22)	<0.01
1990-1999	-1.7 (-1.95,-1.45)	<0.01	-0.74 (-0.98,-0.51)	<0.01	0 (-0.07,0.06)	0.94	0 (-0.05,0.06)	0.92
2000-2009	-2.16 (-3.06,-1.25)	<0.01	-0.49 (-0.75,-0.23)	<0.01	0.73 (0.7,0.76)	<0.01	0.7 (0.67,0.73)	<0.01
2010-2021	-0.92 (-1.16,-0.68)	<0.01	-0.35 (-0.66,-0.05)	0.02	-0.06 (-0.07,-0.05)	<0.01	-0.04 (-0.05,-0.03)	<0.01

#### Global trends by sex

From 1990 to 2019, the global incidence of urolithiasis increased in both males and females, with an AAPC of 0.16 (95% CI 0.13, 0.18). In 1990, the incidence was 367.5 per 100,000 in males and 273.9 per 100,000 in females. However, by 2021, the incidence had risen to 386.1 per 100,000 in males and 296.8 per 100,000 in females. Despite this increase, both males and females experienced a global decline in urolithiasis from 1990 to 2021, with an AAPC of -0.53 in both groups. In 2021, the total global incidence of urolithiasis was 6,467,487 million, with 57.8% of cases occurring in males. 

#### Global trends by age group

The prevalence of urolithiasis has shown significant variations globally across different age groups from 1990 to 2021. Among these age groups, individuals aged 20 – 24 years experienced the most notable increase in incidence, with rates rising from 557.6 per 100,000 population in 1990 to 610.2 per 100,000 population in 2021. This increase corresponds to an Average Annual Percent Change (AAPC) of 0.29. Similarly, the 15 – 19 year age group also saw a rise in urolithiasis incidence, from 279.7 per 100,000 population to 301 per 100,000 population during the same period, with an AAPC of 0.24. In contrast, the 10 – 14 year age group showed a decrease in urolithiasis incidence, from 145.1 per 100,000 population in 1990 to 141.8 per 100,000 population in 2021, with an AAPC of -0.07. The trends in urolithiasis incidence among these age subgroups are summarized in **Table 2**, highlighting the variations seen over the past three decades (**Table 2**).

**Table 2:** The incidence and DALYs of urolithiasis and the AAPCs from 1990 to 2021 at the global and regional levels.

Parameter	Incidence Case (n), 1990	Incidence rate, 1990	Case (n), 2021	Incidence rate, 2021	AAPC (1990-2021)	p VALUE	DALYS Case (n), 1990	DALYS rate, 1990	Case (n), 2021	DALYs rate, 2021	AAPC (1990-2021)	P VALUE
Global	4973653 (2834684,7885655)	321.5 (183.2,509.7)	6467486 (3787933,9862147)	342.6 (200.7,522.4)	0.2 (0.18,0.23)	<0.01	26260 (17997,36785)	1.7 (1.2,2.4)	27346 (18953,39487)	1.4 (1,2.1)	-0.51 (-0.67,-0.35)	<0.01
Low SDI	478576 (279223,733455)	307.4 (179.4,471.2)	1183064 (687683,1816263)	320.3 (186.2,491.7)	0.13 (0.11,0.16)	<0.01	2523 (1514,3642)	1.6 (1,2.3)	5340 (3439,7966)	1.4 (0.9,2.2)	-0.37 (-0.44,-0.29)	<0.01
Middle SDI	1522773 (858332,2450019)	277.5 (156.4,446.4)	1762329 (1034652,2697879)	318.8 (187.2,488.1)	0.45 (0.41,0.49)	<0.01	10047 (6089,13799)	1.8 (1.1,2.5)	7885 (5566,11330)	1.4 (1,2)	-0.81 (-1.02,-0.61)	<0.01
High-middle SDI	999345 (567250,1613365)	352.1 (199.9,568.5)	725437 (433545,1109276)	321.2 (191.9,491.1)	-0.31 (-0.38,-0.25)	<0.01	4900 (3424,6990)	1.7 (1.2,2.5)	2588 (1635,3879)	1.1 (0.7,1.7)	-1.34 (-1.53,-1.14)	<0.01
Low-middle SDI	1295406 (758817,2006265)	358.1 (209.8,554.7)	2260746 (1319764,3483243)	409 (238.8,630.2)	0.43 (0.38,0.48)	<0.01	6398 (3974,9303)	1.8 (1.1,2.6)	9657 (6619,14128)	1.7 (1.2,2.6)	-0.07 (-0.22,0.09)	0.41
High SDI	672592 (381847,1090833)	343.4 (195.0,557.0)	531660 (336020,803573)	286.5 (181.1,433)	-0.59 (-0.81,-0.38)	<0.01	2371 (1463,3654)	1.2 (0.7,1.9)	1861 (1180,2804)	1 (0.6,1.5)	-0.64 (-0.8,-0.47)	<0.01
SEX												
Female	2085030 (1189402,3261378)	273.9 (156.3,428.5)	2731977 (1598130,4162114)	296.8 (173.6,452.2)	0.26 (0.22,0.29)	<0.01	11886 (7563,16627)	1.6 (1,2.2)	12296 (8636,17835)	1.3 (0.9,1.9)	-0.53 (-0.76,-0.3)	<0.01
Male	2888623 (1646980,4605467)	367.5 (209.5,585.9)	3735509 (2192072,5721708)	386.1 (226.6,591.4)	0.16 (0.13,0.18)	<0.01	14374 (9348,20417)	1.8 (1.2,2.6)	15051 (9893,22243)	1.6 (1.0,2.3)	-0.53 (-0.59,-0.48)	<0.01
Age group years												
10-14	777095 (377745,1243562)	145.1 (70.5,232.1)	945124 (471926,1479604)	141.8 (70.8,222)	-0.07 (-0.11,-0.04)	<0.01	4319 (2725,6185)	0.8 (0.5,1.2)	3928 (2463,6020)	0.6 (0.4,0.9)	-0.97 (-1.13,-0.82)	<0.01
15-19	1452755 (756915,2459892)	279.7 (145.7,473.6)	1878382 (1019878,3077858)	301 (163.4,493.3)	0.24 (0.21,0.27)	<0.01	7402 (4816,10910)	1.4 (0.9,2.1)	7512 (4891,11509)	1.2 (0.8,1.8)	-0.53 (-0.59,-0.47)	<0.01
20-24	2743804 (1495868,4739609)	557.6 (304,963.2)	3643981 (2070256,6108622)	610.2 (346.7,1022.9)	0.29 (0.24,0.34)	<0.01	14539 (9275,20967)	3 (1.9,4.3)	15907 (10617,23295)	2.7 (1.8,3.9)	-0.35 (-0.43,-0.27)	<0.01
Region												
East Asia	797198 (427574,1369442)	214.2 (114.9,368)	340644 (200461,538645)	140.2 (82.5,221.7)	-1.37 (-1.51,-1.24)	<0.01	6812 (3482,9586)	1.8 (0.9,2.6)	1583 (1068,2264)	0.7 (0.4,0.9)	-3.31 (-3.6,-3.02)	<0.01
Southeast Asia	319587 (176905,523890)	215.4 (119.2,353.1)	374360 (220278,591480)	218.9 (128.8,345.9)	0.06 (0.03,0.09)	<0.01	2486 (1362,3460)	1.7 (0.9,2.3)	2420 (1370,3314)	1.4 (0.8,1.9)	-0.53 (-0.69,-0.37)	<0.01
Oceania	3595 (1978,5844)	171.8 (94.6,279.4)	7458 (4124,11766)	184.9 (102.3,291.8)	0.24 (0.22,0.26)	<0.01	10 (5,18)	0.5 (0.2,0.8)	21 (11,36)	0.5 (0.3,0.9)	0.22 (0.2,0.24)	<0.01
Eastern Europe	394844 (237490,611123)	836 (502.9,1294)	264653 (158812,404988)	802.1 (481.3,1227.4)	-0.15 (-0.21,-0.08)	<0.01	1669 (1124,2389)	3.5 (2.4,5.1)	949 (595,1418)	2.9 (1.8,4.3)	-0.68 (-1.02,-0.33)	<0.01
Australasia	11027 (6022,18473)	229.2 (125.2,384)	12970 (7357,21583)	226.1 (128.2,376.2)	-0.04 (-0.11,0.02)	0.2	35 (19,57)	0.7 (0.4,1.2)	38 (20,64)	0.7 (0.4,1.1)	-0.31 (-0.43,-0.19)	<0.01
Central Europe	182925 (109107,279903)	626.5 (373.7,958.6)	102494 (65141,148338)	565.1 (359.2,817.9)	-0.32 (-0.38,-0.27)	<0.01	754 (516,1094)	2.6 (1.8,3.7)	311 (185,480)	1.7 (1,2.6)	-1.29 (-1.45,-1.13)	<0.01
Central Asia	131585 (78758,202039)	663.5 (397.1,1018.8)	148440 (89667,227320)	670.8 (405.2,1027.3)	0.02 (-0.06,0.1)	0.61	477 (297,718)	2.4 (1.5,3.6)	489 (293,758)	2.2 (1.3,3.4)	-0.29 (-0.59,0.01)	0.06
High-income Asia Pacific	125900 (71598,203133)	298.9 (170,482.2)	69046 (41275,106954)	264.5 (158.1,409.7)	-0.4 (-0.45,-0.34)	<0.01	394 (222,649)	0.9 (0.5,1.5)	235 (144,363)	0.9 (0.6,1.4)	-0.1 (-0.28,0.09)	0.31
Andean Latin America	41376 (24209,65613)	336.1 (196.7,533)	64340 (41346,97511)	372.7 (239.5,564.8)	0.34 (0.31,0.36)	<0.01	146 (91,217)	1.2 (0.7,1.8)	223 (144,333)	1.3 (0.8,1.9)	0.26 (0.06,0.45)	0.01
Caribbean	25797 (14739,41547)	241.6 (138,389.1)	28109 (16030,45340)	248.2 (141.5,400.3)	0.08 (0.06,0.11)	<0.01	85 (49,136)	0.8 (0.5,1.3)	101 (63,161)	0.9 (0.6,1.4)	0.39 (0.23,0.55)	<0.01
Western Europe	216811 (121772,357642)	263.8 (148.1,435.1)	184268 (112403,285603)	255.7 (156,396.3)	-0.1 (-0.26,0.06)	0.23	785 (486,1203)	1 (0.6,1.5)	608 (363,941)	0.8 (0.5,1.3)	-0.4 (-0.52,-0.29)	<0.01
Southern Latin America	52075 (30621,84788)	393.4 (231.3,640.5)	69752 (44638,105272)	454.8 (291.1,686.4)	0.46 (0.42,0.51)	<0.01	149 (79,244)	1.1 (0.6,1.8)	205 (118,323)	1.3 (0.8,2.1)	0.54 (0.42,0.66)	<0.01
High-income North America	265297 (151908,427882)	433.7 (248.3,699.5)	225166 (148715,319134)	315.9 (208.6,447.7)	-1.08 (-1.31,-0.86)	<0.01	859 (495,1357)	1.4 (0.8,2.2)	841 (569,1207)	1.2 (0.8,1.7)	-0.59 (-0.85,-0.33)	<0.01
Tropical Latin America	74247 (45222,114151)	155.1 (94.5,238.5)	149917 (93004,224326)	296.4 (183.9,443.5)	2.07 (1.9,2.24)	<0.01	423 (323,555)	0.9 (0.7,1.2)	1239 (1030,1511)	2.5 (2,3)	3.44 (3.12,3.75)	<0.01
Central Latin America	117839 (67289,188529)	217.2 (124,347.5)	149814 (87048,229660)	230.4 (133.9,353.1)	0.2 (0.06,0.33)	<0.01	776 (619,1015)	1.4 (1.1,1.9)	881 (678,1180)	1.4 (1,1.8)	-0.17 (-0.51,0.18)	0.35
North Africa and Middle East	290665 (168833,453757)	266.9 (155,416.6)	454223 (263466,704297)	279.9 (162.3,434)	0.15 (0.12,0.18)	<0.01	1335 (880,1942)	1.2 (0.8,1.8)	1820 (1150,2740)	1.1 (0.7,1.7)	-0.28 (-0.37,-0.2)	<0.01
South Asia	1511289 (896113,2328928)	451.8 (267.9,696.3)	2836816 (1678879,4400105)	539.5 (319.3,836.7)	0.57 (0.52,0.62)	<0.01	6838 (3946,9965)	2 (1.2,3)	10488 (6639,15662)	2 (1.3,3)	-0.13 (-0.35,0.09)	0.25
Western Sub-Saharan Africa	144911 (83284,224481)	242.1 (139.2,375.1)	395083 (226118,612447)	244.8 (140.1,379.5)	0.05 (0.01,0.08)	<0.01	727 (448,1112)	1.2 (0.7,1.9)	1835 (1114,2901)	1.1 (0.7,1.8)	-0.22 (-0.25,-0.19)	<0.01
Southern Sub-Saharan Africa	57275 (33582,87394)	335.3 (196.6,511.6)	77422 (45110,117950)	354.9 (206.8,540.7)	0.2 (0.16,0.23)	<0.01	194 (114,305)	1.1 (0.7,1.8)	271 (168,414)	1.2 (0.8,1.9)	0.31 (0.17,0.45)	<0.01
Central Sub-Saharan Africa	39883 (23148,60975)	230.4 (133.7,352.3)	105773 (60602,162519)	235.3 (134.8,361.6)	0.07 (0.05,0.09)	<0.01	140 (80,220)	0.8 (0.5,1.3)	357 (208,570)	0.8 (0.5,1.3)	-0.06 (-0.09,-0.04)	<0.01
Eastern Sub-Saharan Africa	169528 (98135,259065)	273.3 (158.2,417.6)	406740 (236305,622177)	279.7 (162.5,427.8)	0.08 (0.06,0.1)	<0.01	1168 (615,2149)	1.9 (1,3.5)	2430 (1451,4208)	1.7 (1,2.9)	-0.37 (-0.46,-0.29)	<0.01

#### Global trends by SDI

The incidence of Urolithiasis has been steadily increasing in countries with middle and low-middle socio-demographics. Specifically, the middle-SDI quintile saw the greatest rise, with rates climbing from 277.5 per 100,000 population in 1990 to 318.8 per 100,000 population in 2021. This trend is significant, reflecting a need for increased awareness and prevention efforts in these regions to address this growing health issue.

The incidence of urolithiasis has shown a decreasing trend in both high-middle and high SDI countries over the years. High SDI countries, in particular, have experienced the most significant reduction in urolithiasis incidence, with numbers dropping from 343.4 per 100,000 population in 1990 to 286.5 per 100,000 population in 2021. This positive trend is reflected in all SDI subgroups, with a noticeable decrease in Disability-Adjusted Life Years (DALYs) attributed to urolithiasis between 1990 and 2021. The High-middle SDI region stands out for its substantial decrease in DALYs, demonstrating an Average Annual Percentage Change (AAPC) of -1.34 between 1990 and 2021. These findings suggest a positive impact on public health interventions and management strategies for urolithiasis (**Table 2**).

### Regional trends

The prevalence of urolithiasis, also known as kidney stones, has been on the rise in Tropical Latin America over the past three decades. From 1990 to 2021, the incidence of this condition increased significantly from 155.1 cases per 100,000 population to 296.4 cases per 100,000 population. This upward trend is also mirrored in South Asia and Southern Latin America, where the incidence of urolithiasis has been steadily climbing. Conversely, East Asia witnessed a notable decrease in the prevalence of urolithiasis during the same period. The incidence rate dropped from 214.2 cases per 100,000 individuals in 1990 to 140.2 cases per 100,000 individuals in 2021. Similar declines were seen in Eastern Europe, Central Europe, High-income North America, and High-income Asia Pacific. These contrasting trends highlight the varied landscape of urolithiasis prevalence across different regions. While some areas are experiencing a concerning increase in the incidence of kidney stones, others are showing promising declines. Understanding these regional differences is crucial for effective prevention and management strategies to combat the burden of urolithiasis on public health. It is essential for healthcare providers and policymakers to address the factors driving the rise of this condition in certain regions while also celebrating the progress made in others. By tailoring interventions to specific regional needs, we can work towards reducing the impact of urolithiasis worldwide.

### National trends

**Figure 2:**
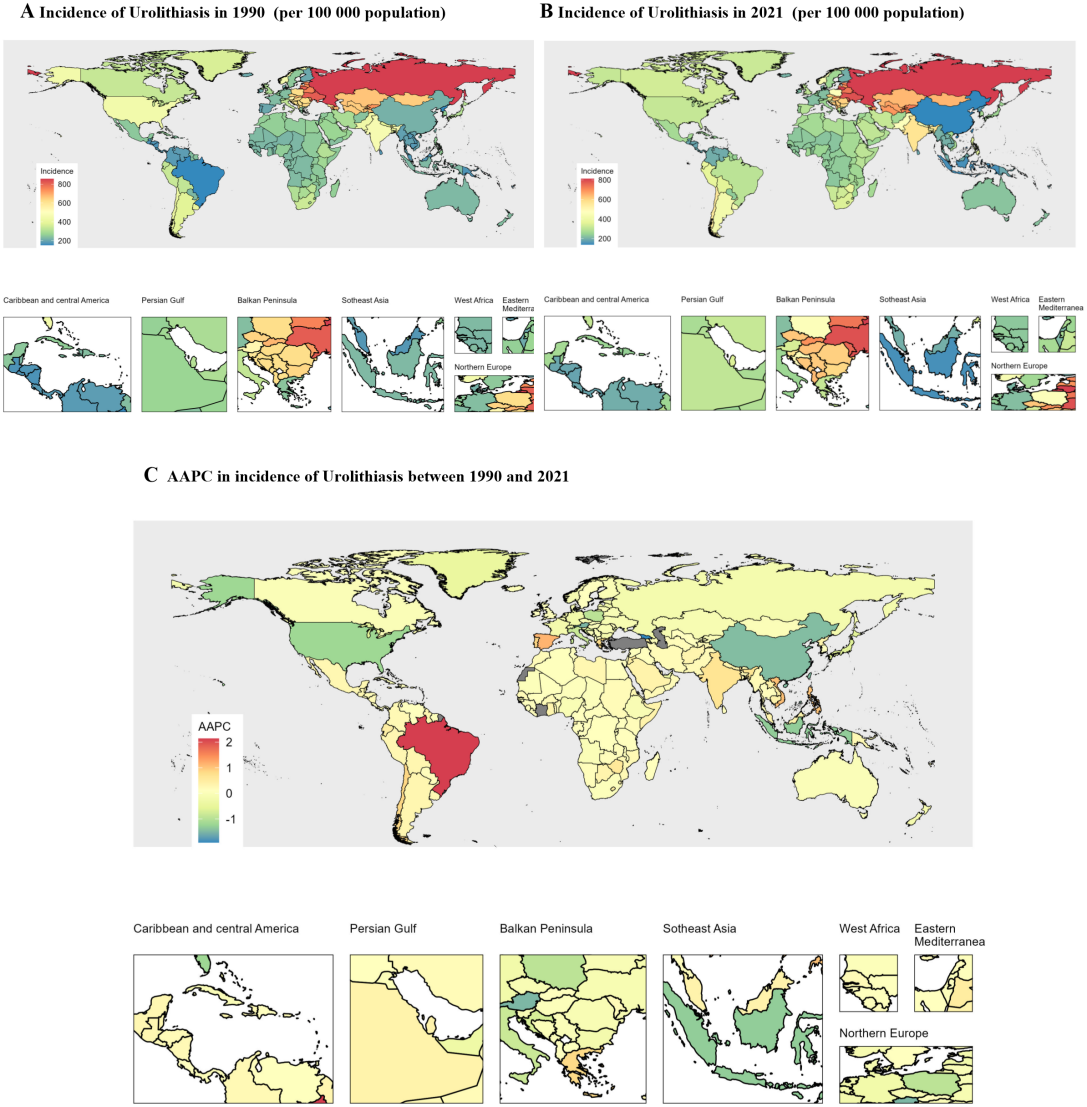
Global map of 1990 incidence of Urolithiasis **(A)** and 2021 incidence of Urolithiasis **(B)** and average annual percentage changes in incidence of Urolithiasis **(C)** from 1990 to 2021. AAPC, average annual percentage change; STI, sexually transmitted infection.

Brazil has shown a significant increase in the prevalence of urolithiasis from 1990 to 2021, with rates rising from 153.2 to 298.8 per 100,000 population. This represented the largest escalation among all countries analyzed, with an Annual Percentage Change (AAPC) of 2.14. The Russian Federation, on the other hand, had the highest incidence of urolithiasis in 2021, with rates reaching 812.7 per 100,000 population. Additionally, Brazil saw a substantial increase in Disability-Adjusted Life Years (DALYs) due to urolithiasis during the same period, with rates rising from 0.9 to 2.5 per 100,000 population. This increase had an AAPC of 3.49. In contrast, the Russian Federation had the highest DALYs attributed to urolithiasis in 2021, at 3 per 100,000 people. These findings highlight the growing burden of urolithiasis in Brazil and the Russian Federation, underscoring the importance of addressing this issue in global health initiatives (**Figure 2**).

## Discussion

This pioneering study examined the prevalence and temporal trends of urolithiasis in individuals aged 10 – 24 years across 204 countries between 1990 and 2019, providing global, regional, and national insights. There has been an overall increase in the global incidence of urolithiasis between 1990 and 2021, although there has been a year-on-year decrease since 1995. The incidence of urinary stones in adolescents increased annually from 2000 to 2010. This may, in part, be attributed to enhanced detection of urinary calculi and subsequent surgical interventions or surveillance (6). The observed reduction in mortality and DALYs rates exhibits temporal alignment with the broad adoption of minimally invasive procedures for urolithiasis management. Our analysis revealed that the decade characterized by the most pronounced decrease in urinary stone-related mortality coincided with a period of significant advancements in minimally invasive surgical techniques. This era has witnessed the sequential introduction and utilization of diverse minimally invasive techniques, including percutaneous nephrolithotomy, ureteroscopy, and other advanced technologies. The period from 1990 to 2010 was marked by recurrent updates to numerous health guidelines with a particular emphasis on dietary recommendations. China inaugurated its “Chinese Dietary Guideline” in 1989, followed by subsequent revisions in 1997, 2007, and 2016.The 2007 edition of the dietary guidelines expanded upon the 1997 recommendations for regular consumption of milk, legumes, and their derivatives by incorporating directives for daily water intake and sodium limitations. Additionally, it underscored the significance of enhanced physical activity in improving nutritional status and reducing or preventing chronic disease incidence (7). Research has indicated a strong association between dietary habits, lifestyle choices, and development of urolithiasis (8). Urinary sepsis was established as the main cause of mortality, representing 49.5% of deaths, while cardiac events were responsible for 20% of fatalities. Notably, 39% (37/95) of deaths were linked to CMIs, with the most prevalent factors being diagnostic or treatment delays, perioperative management issues, and insufficient preoperative assessment (9). Nephrolithiasis is a life-threatening condition, and mortality rates associated with its treatment are considered minimal. The decline in DALYs and mortality over the past 30 years is strongly associated with the development of guidelines, particularly since the introduction of SOFA in 1991 and its subsequent inclusion in the guidelines, providing an effective diagnostic tool for the rapid diagnosis of urosepsis (10). In 1991, a collaborative effort between the American College of Chest Physicians and Society of Critical Care Medicine formed a concurrence panel. This panel was tasked with formulating recommendations to enhance the clarity and precision of sepsis definition. The conceptual framework includes SIRS, sepsis, severe sepsis, and septic shock. In 2001, a concurrence panel revised and expanded these definitions. The Sepsis-3 Task Force made the latest modifications to the definition of sepsis in 2016. The authors described sepsis as a critical organ dysfunction resulting from an abnormal immune response to infection. The authors recommended using the Sequential Organ Failure Assessment (SOFA) to evaluate organ dysfunction in sepsis. Given SOFA’s limited accessibility of SOFA, which requires blood gas measurement for PaO2, researchers introduced the quick Sequential Organ Failure Assessment (qSOFA) for the rapid medical assessment of sepsis risk (11).

While urolithiasis remains more prevalent in males globally, from 1990 to 2021, the Average Annual Percent Change (AAPC) is higher in females (0.26 [95% CI 0.22, 0.29]) than in males (0.16 [95% CI 0.13, 0.18]). Although the male-to-female ratio of urolithiasis has declined from 3:1 to approximately 2:1 over the past two decades (12), our research found that the male-to-female ratio of urolithiasis among adolescents appears to be 1.3:1, which is lower than the global ratio, suggesting that the incidence of urolithiasis among adolescents tends to be similar in both sexes. In some studies, we observed sex differences in the incidence of urolithiasis. This finding is congruent with those of previous investigations. This disparity may be related to different nutritional habits. Men are more likely to ingest higher quantities of protein and alcohol than women are. The incidence of urolithiasis in both sexes showed an age-dependent pattern, initially increasing and then declining after peaking (7, 13). Research indicates a potential link between stone composition and sex, with females showing a higher prevalence of hydroxyapatite and struvite calculi. Struvite and carbonate-apatite stones, known as ‘infection stones,’ are associated with a higher risk of urosepsis (12). This may explain the observed sex disparities in urolithiasis-related mortality rates.

The study found the highest incidence of urolithiasis in the older age groups throughout the study period. The incidence of urolithiasis among individuals aged 20 – 24 years was 601.2 per 100,000 people, which was more than four times that observed among adolescents aged 10 – 14 years (141.8 per 100,000 population). The incidence among those aged 15 – 19 years was 301 per 100,000 people, more than twice that observed among juveniles aged 10 – 14 years. However, the 10 – 14 years age group was the only cohort that exhibited a significant decrease in the incidence of urolithiasis between 1990 and 2021. This phenomenon may be associated with age-related dietary behaviors, including alcohol consumption, tobacco use, and reduced fluid intake (13). Our findings indicate that the type of urolithiasis with the highest incidence varies according to age, suggesting that age may be utilized as a factor to facilitate targeted urolithiasis screening.

Regionally, adolescents in the Low-SDI and Low-Middle SDI groups accounted for 3,443,810 (53.2%) of the 6,467,486 global urolithiasis incidences in this population in 2021. This may be attributed to the fact that these regions often lack resources to provide a high-income country (HIC) standard of care at governmental or institutional levels. Furthermore, in countries without universal health coverage (UHC), the impact on individuals is substantial (14). The prevalence of urinary stones demonstrates a correlation with socioeconomic disparities, revealing not only variations in stone incidence between affluent and economically disadvantaged regions, but also marked differences in the anatomical site of stone formation. Notably, upper urinary tract stones are predominantly observed in high-income areas, whereas lower urinary tract stones are more commonly encountered in low-income regions such as the Sahara Desert in Africa (14). Nevertheless, this phenomenon may also be attributed to the geographical distribution of low-income countries, predominantly in tropical regions, as the incidence of stones in these areas reaches 10%, which is significantly higher than that observed in other Asian countries (15). In 2021, the regions encompassing Southeast Asia, East Asia, Central Asia, High-income Asia Pacific, and South Asia collectively represented 3,768,306 (58.3%) of the 6,467,486 adolescents diagnosed with urolithiasis worldwide. Notably, three nations-India, China, and the Russian Federation–accounted for approximately half of the worldwide incident cases reported in 2019 (16). However, this phenomenon was also closely correlated with the population distribution in these regions. China and India are the world’s most populous and second-most populous nations, respectively. Although China accounted for a substantial proportion of the number of patients, the incidence rate in 2021 was the lowest globally, at 138.3 (95%UI 81.4,218.7). According to the global chart (**Figure 2**), the incidence rate in 1990 was 216.1 (95%UI 115.7,371.2), and the AAPC was -1.45 (95%CI -1.59, -1.3). The decrease in the ASIR of urolithiasis in China may be attributed to recent changes in China’s dietary composition. Over the past two decades, the intake of fruit and plant-based foods by young Chinese people and adults has increased (7). From 1990 to 2021, Russia consistently had the highest stone prevalence rate. Research shows a simultaneous rise in the incidence of urolithiasis with increasing rates of diabetes mellitus, obesity, and meat consumption, highlighting the significant role of metabolic syndrome in urinary stone formation. The highest incidence of urolithiasis was observed in Siberia and the Far East. This study suggests a link between elevated urolithiasis prevalence and local dietary patterns, as indicated by the correlation between urolithiasis incidence and diabetes mellitus, obesity, and meat consumption (17).

The highest surges in urolithiasis incidence between 1990 and 2021 were noted in Tropical Latin America, rising from 155.1 per 100,000 population in 1990 to 296.4 per 100,000 population in 2021; AAPC 2.07 (95%CI 1.9,2.24). This region also exhibited the highest Urolithiasis DALYs rate between 1990 and 2021, increasing from 0.9 per 100,000 population in 1990 to 2.5 per 100,000 population in 2021; AAPC 3.44(95%CI 3.12,3.75). Studies have shown that cola consumption contributes to urolithiasis (18). According to a 2021 report, Brazil and numerous other countries in Tropical Latin America exhibit the highest annual consumption of cola beverages, which may account for the substantial growth in the incidence of urolithiasis in these regions (19). The etiology of urolithiasis and calcium kidney stones in adults has been attributed to several key risk factors including inadequate fluid consumption, obesity, extended life expectancy, global climate change, and excessive sodium intake (20, 21). Notably, East Asia demonstrated the most significant reduction in urolithiasis incidence from 1990 to 2021, with rates decreasing from 214.2 to 140.2 per 100,000 population and the AAPC was -1.37 (95%CI -1.51,-1.24). Similarly, this region experienced the most substantial decline in urolithiasis DALYs rate, dropping from 1.8 to 0.7 per 100,000 population during the same period and the AAPC was -3.31(95%CI -3.5,-3.02). Diverse climates and cultures across Asia present challenges in accurately characterizing the prevalence and incidence of urolithiasis. However, Japan, China, and Korea have reported upward trends in stone formation. In China, coastal provinces in the south exhibit the highest rates of urinary stones, a phenomenon attributed to the elevated calcium/magnesium ratios in drinking water and proximity to phosphate mines and carbonate rocks. The observed decrease in urolithiasis AAPC over the past three decades can be largely attributed to advancements in surgical techniques and evolution of clinical guidelines (1, 22).

The results of this study have implications for future research. A report from 2024 revealed a continued upward trend in the worldwide prevalence of urolithiasis in recent decades (23). This study’s comprehensive analysis of the burden associated with the three urological benign diseases across various global regions and countries is not without limitations. Primarily, the restricted nature of GBD data sources precludes the complete coverage of all populations and regions, resulting in data that merely represent the general circumstances of specific areas. Additionally, inconsistencies in data quality exist, with potential heterogeneity stemming from disparities in diagnostic criteria, detection methodologies, and monitoring systems among regions at different developmental stages. Constraints in the definition of GBD may contribute to underestimation of the true disease burden.

## Conclusion

Urolithiasis among adolescents is a substantial global public health challenge. Addressing this issue requires policymakers to account for the growing number of individuals impacted by urological disorders and the consequences of an aging population. Such considerations are crucial for fostering international cooperation aimed at enhancing the well-being of those suffering from urological diseases. This collaborative effort should encompass the creation of efficient diagnostic screening methods and execution of superior prevention and treatment approaches.

## Data Availability

The datasets presented in this study can be found in online repositories. The names of the repository/repositories and accession number(s) can be found in the article/supplementary material.

## References

[1] Raheem OA Khandwala YS Sur RL Ghani KR Denstedt JD . Burden of urolithiasis: trends in prevalence, treatments, and costs. Eur Urol Focus. (2017) 3:18–26. doi: 10.1016/j.euf.2017.04.001, PMID: 28720363

[2] Zi H Li M-Y Luo L-S Huang Q Luo P-C Luan H-H . Global burden of benign prostatic hyperplasia, urinary tract infections, urolithiasis, bladder cancer, kidney cancer, and prostate cancer from 1990 to 2021. Mil Med Res. (2024) 11:64. doi: 10.1186/s40779-024-00569-w, PMID: 39294748 PMC11409598

[3] Habbig S Beck BB Hoppe B . Nephrocalcinosis and urolithiasis in children. Kidney Int. (2011) 80:1278–91. doi: 10.1038/ki.2011.336, PMID: 21956187

[4] WHO . Adolescent health . Available online at: https://www.who.int/health-topics/adolescent-healthtab=tab_1 (Accessed June 18, 2022).

[5] Sawyer SM Azzopardi PS Wickremarathne D Patton GC . The age of adolescence. Lancet Child Adolesc Health. (2018) 36:S2352464218300221. doi: 10.1016/S2352-4642(18)30022-1, PMID: 30169257

[6] Kum F Mahmalji W Hale J Thomas K Bultitude M Glass J . Do stones still kill? An analysis of death from stone disease 1999–2013 in England and Wales. Bju Int. (2016) 15:e914–e. doi: 10.1016/S1569-9056(16)60916-8, PMID: 26765522

[7] Huang H Li M Fan H Bai R . Temporal trend of urolithiasis incidence in China: an age–period–cohort analysis. Int J Gen Med. (2021) 14:2533–9. doi: 10.2147/IJGM.S313395, PMID: 34163221 PMC8214536

[8] Eisner BH Goldfarb DS . A nomogram for the prediction of kidney stone recurrence. J Am Soc Nephrol JASN. (2014) 25:2685–7. doi: 10.1681/ASN.2014060631, PMID: 25104802 PMC4243365

[9] Galiabovitch E Hansen D Retegan C McCahy P . Urinary tract stone deaths: data from the Australian and New Zealand Audits of Surgical Mortality. BJU Int. (2020) 126:604–9. doi: 10.1111/bju.15171, PMID: 32654379

[10] Ryoo SM Kang GH Shin TG Hwang SY Kim WY . Clinical outcome comparison of patients with septic shock defined by the new sepsis-3 criteria and by previous criteria. J Thorac Dis. (2018) 10:845. doi: 10.21037/jtd.2018.01.96, PMID: 29607156 PMC5864585

[11] Pandey S Sankhwar SN Goe A Kumar M Aggarwal A Sharma D . Quick Sequential (Sepsis Related) Organ Failure Assessment: A high performance rapid prognostication tool in patients having acute pyelonephritis with upper urinary tract calculi. Invest Clin Urol. (2019) 60:446–53. doi: 10.4111/icu.2019.60.2.120, PMID: 30838345 PMC6397933

[12] Lieske JC Rule AD Krambeck AE Williams JC Bergstralh EJ Mehta RA . Stone composition as a function of age and sex. Clin J Am Soc Nephrol Cjasn. (2014) 9:2141–6. doi: 10.2215/CJN.05660614, PMID: 25278549 PMC4255407

[13] Liu Y Chen Y Liao B Luo D Wang K Li H . Epidemiology of urolithiasis in asia. Asian J Urol. (2018) 5:205–9. doi: 10.1016/j.ajur.2018.08.007, PMID: 30364478 PMC6197415

[14] Watson G Payne SR Kunitsky K Natchagande G Mabedi C Scotland KB . Stone disease in low- and middle-income countries: could augmented reality have a role in its management? BJU Int. (2022) 130:400–7. doi: 10.1111/bju.15877, PMID: 35993671

[15] Dawson CH Tomson CR . Kidney stone disease: pathophysiology, investigation and medical treatment. Clin Med. (2012) 12:467. doi: 10.7861/clinmedicine.12-5-467, PMID: 23101150 PMC4953772

[16] JL A AN B EZA C PSA C . C OEA. Global Trends Incidence Burden Urolithiasis 1990 to 2019: Anal Global Burden Dis Study Data. Eur Urol Open Sci. (2022) 35:37–46. 10.1016/j.euros.2021.10.008 PMC8738898 35024630

[17] Prosyannikov NGM . Urolithiasis prevalence in the Russian Federation: analysis of trends over a 15-year period. World J Urol. (2021) 2021:1–6., PMID: 34008087 10.1007/s00345-021-03729-y

[18] Rodgers A . Effect of cola consumption on urinary biochemical and physicochemical risk factors associated with calcium oxalate urolithiasis. Urol Res. (1999) 27:77–81. doi: 10.1007/s002400050092, PMID: 10092157

[19] Carbonated soft drink market size, share & Trends analysis report by flavor (Cola, citrus), by distribution channel (Hypermarkets, supermarkets & Mass merchandisers, online stores & D2C), and segment forecasts, 2021-2028. In: Research GV. (Ireland: Research and Markets)

[20] Ciongradi CI Filip F Sarbu I Iliescu Halitchi CO Munteanu V Candussi IL . The impact of water and other fluids on pediatric nephrolithiasis. Nutrients. (2022) 14:4161. doi: 10.3390/nu14194161, PMID: 36235817 PMC9573375

[21] Romero V Akpinar H Assimos DG . Kidney stones: a global picture of prevalence, incidence, and associated risk factors. Rev Urol. (2010) 12:e86., PMID: 20811557 PMC2931286

[22] Zhu C Wang DQ Zeng HZH-MG-YL-PGLF-DL-T . Epidemiological trends of urinary tract infections, urolithiasis and benign prostatic hyperplasia in 203 countries and territories from 1990 to 2019. military Med Res. (2021) 8:64–75. doi: 10.1186/s40779-021-00359-8, PMID: 34879880 PMC8656041

[23] Skolarikos A Somani B Neisius A Jung H Petrík A Tailly T . Metabolic evaluation and recurrence prevention for urinary stone patients: an EAU guidelines update. Eur Urol. (2024) 86:343–63. doi: 10.1016/j.eururo.2024.05.029, PMID: 39069389

